# Improvement in Patient-Reported Quality of Life Outcomes in Severely Visually Impaired Individuals Using the Aira Assistive Technology System

**DOI:** 10.1167/tvst.7.5.30

**Published:** 2018-10-29

**Authors:** Brian J. Nguyen, Yeji Kim, Kathryn Park, Allison J. Chen, Scarlett Chen, Donald Van Fossan, Daniel L. Chao

**Affiliations:** 1 Shiley Eye Institute, Department of Ophthalmology, University of California, San Diego, La Jolla, CA, USA

**Keywords:** wearable technology, severe vision impairment, adaptive technology

## Abstract

**Purpose:**

We evaluate patient-reported quality of life outcomes in severely visually impaired (SVI) individuals using the Aira system, an on demand assistive wearable technology.

**Methods:**

Aira is an on-demand assistive wearable technology designed for the severely visually impaired (visual acuity of better eye <20/200). The user wears glasses with a video camera mounted that, when activated, livestreams to a human agent who assists the user in the specified task. Aira subscribers were recruited consecutively and administered the 28-item Impact of Vision Impairment-Very Low Vision (IVI-VLV) Questionnaire, a previously validated survey for vision-related quality of life specifically for low vision individuals. The questionnaire was administered by phone before starting Aira and at 3-month follow-up. Total score as well as validated subset scores of activities of daily living, mobility and safety (ADLMS) and emotional wellbeing (EWB) were assessed.

**Results:**

A total of 69 participants (mean age, 52.1; 35 female, 34 male) were recruited with a mean of 108 (SD = 19.7) days to follow-up. Mean total minutes used over the interval were 334.1 (SD = 318.5). Initial total score (mean 51.7 ± 18.6) significantly improved at follow-up (mean 62.2 ± 15.0; *P* < 0.0001) with mean change +10.4 ± 12.5. ADLMS subset score (mean 30.4 ± 10.8) significantly improved at follow-up (mean 36.6 ± 8.8; *P* < 0.0001) with mean change +6.5 ± 8.7. EWB subset score (mean 21.6 ± 8.8) significantly improved at follow-up (mean 25.6 ± 7.9 respectively; *P* < 0.0001) with mean change +4.0 ± 5.2. There was no correlation between minutes used and improvement in total (*r* = −0.205, *P* = 0.098), ADLMS (*r* = −0.237, *P* = 0.055), and EWB (*r* = −0.242, *P* = 0.051) scores.

**Conclusions:**

In this exploratory study, regardless of minutes used, the use of Aira significantly improves IVI-VLV total score and ADLMS and EWB subscores for SVI individuals. This improvement is not correlated with total minutes used.

**Translational Relevance:**

The Aira assistive technology system may provide improvement in quality of life for low vision patients and is worthy of further study to assess the use of this technology to assist SVI patients.

## Introduction

Severe vision impairment (SVI), defined as vision less than 20/200 in the better eye, and blindness are disabling conditions with multiple etiologies, including congenital disorders, such as Leber congenital amaurosis, retinitis pigmentosa and pathologic processes such as untreated diabetic retinopathy, trauma, glaucoma, and retinal detachment. Vision impairment affects over 250 million people worldwide with 36 million of those completely blind, resulting in extensive socioeconomic impacts as well as personal psychologic and quality of life deficits.^1^ Though there is a paucity of epidemiologic data describing varying degrees of vision impairment in the United States, recent studies of adults aged 40 and older estimate prevalences of up to 2.14% of for uncorrectable SVI and 0.86% for blindness.^2,3^ In the United States, SVI is one of 10 leading reported disabilities of adults aged 18 and older.^4^ With current population growth patterns expected to shift into a right-modal distribution with rapidly expanding geriatric demographics and continued chronic disease epidemics, such as diabetes, prevalence of debilitating eye disease potentially leading to SVI is estimated to double by 2050.^2^ The economic burden of SVI for Americans aged 40 and older totals over 35 billion dollars with 8 billion stemming from productivity losses.^5^

In patients with untreatable severe visual impairment, various low vision assistive aids, such as guide dogs, walking canes, Braille, and various magnifiers, currently are used to improve function. However, little rigorous clinical data exist demonstrating the use of these various low vision interventions. A major need in the low vision field is the validation of emerging low vision technologies in improving quality of life for these patients. As visual acuity is not a reliable endpoint in these patients who have very poor vision or may be completely blind, patient-reported outcomes may serve as the best way to assess the ability of low vision assistive technologies to improve quality of life in this population.

Patient-reported outcomes have become increasingly important in evaluation of new investigational medical devices and therapeutics. Self-reported patient outcomes are a common secondary endpoint for ophthalmology clinical trials, with the National Eye Institute Visual Function Questionnaire 25 (NEI VFQ-25) as the most common and widely used questionnaire.^6^ However, the VFQ-25 does not accurately assess quality of life in patients with severe visual acuity as it was not designed or validated in low vision patients. Additionally, it does not use Rasch analysis and, thus, does not accurately address psychometric measures that are nonlinear in raw form.^7,8^ Rasch analysis is a mathematical modeling technique for categorical data that allows nonlinear raw data to be converted to a linear scale, which then can be understood with statistical tests. The model assumes the interaction between the person and the item is only determined by item difficulty and person ability. The result is equivalent spread of items, increased precision, reduction of noise, and simplicity of data.^9^

The Impact of Vision Impairment-Very Low Vision (IVI-VLV) Questionnaire recently has been developed and validated in those with severe vision impairment to assess quality of life and was designed to serve as a questionnaire to assess visual acuity after retinal prosthesis implantation in blind individuals.^10^ This questionnaire benefits from structured Rasch analysis built into the rubric to optimize questions and response levels.^10^

**Figure 1:**
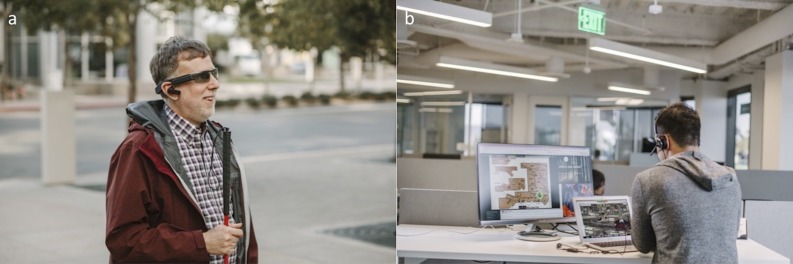
(a) Severely visually impaired individual using Aira with livestreaming to (b) agent with module consisting of the device's video view and applications that assist with task coordination. Pictures courtesy of Aira.

Aira (available in the public domain at https://aira.io/; Aira Tech Corporation, La Jolla, CA) is an on-demand assistive wearable technology designed for the severely visually impaired.^11^ Serving as an augmented reality (AR) conduit for those with SVI, the user wears glasses with a video camera mounted that, when activated, livestreams to an “agent” who assists the user in the specified task. The agent's module consists of the livestream and applications, such as maps that provide further real-time tracking. (Fig. 1) The service is available from 4 AM to 10 PM Pacific Daylight Time. The user is linked to one of 125 agents based on availability. Agents are trained in appropriate low vision communication skills in an intensive course that lasts approximately 2 weeks through a mixture of online-, text-, and video-based modules as well as real-time group training sessions with an agent management team, as well as visually impaired users. Since to our knowledge no studies have examined the efficacy of this novel form of assistive technology, the purpose of this exploratory prospective study was to assess patient-reported quality of life outcomes of SVI individuals after using the Aira system.

## Materials and Methods

### Patients

Individuals, also termed Aira “explorers,” who purchased a subscription to the Aira system were consecutively recruited from December 2016 to December 2017 to participate in this study. The inclusion criteria were severe vision impairment, defined as visual acuity of better eye <20/200; agreement to complete an IVI-VLV Questionnaire; and age 18 and older. There were four monthly subscription levels: 100 minutes ($89 USD), 200 minutes ($129 USD), 400 minutes ($199 USD), and unlimited minutes ($329 USD).^11^ This Health Information Portability and Accountability Act (HIPAA)–compliant study was approved by the University of California, San Diego institutional review board and was conducted in accordance with the principles of the Declaration of Helsinki. All participants provided verbal consent by telephone after an approved verbal script consent was administered. Demographic information such as age, sex, and educational level was collected.

### Assessment of Impact of Vision Impairment

Before activating their Aira subscription, patients were contacted by phone and were verbally administered the IVI-VLV questionnaire^10^ (Supplementary Fig. S1). All patients were English-speaking and were asked to complete the questionnaires, with questions divided into four sections: activities of daily living, mobility, safety, and emotional well-being. The subcategories of activities of daily living, mobility, and safety (ADLMS); emotional well-being (EWB); and total scores have been validated previously.^10,12^ The questions asked the participant to analyze how vision affected one of the four axes within the past month. The Likert grading scale was used with the following possible scores and responses: (0) “a lot,” (1) “sometimes,” (2) “a little,” (3) “not at all,” (8) “don't do this for other reasons.” Sums of scores were calculated for each section with higher scores indicating higher quality of life. Follow-up by phone was completed at 3 months with the same IVI-VLV questionnaire asked in identical order by the same person who administered the initial evaluation. Total minutes used during the 3-month interval were logged internally in Aira databases. Preliminary cost analysis was performed against a prior study by Wirth and Rein, which analyzed the cost-benefits of a guide dog.^13^

### Statistical Analysis

Pearson correlation coefficients and *P* values were calculated between the minutes and improvement in IVI-VLV total score, ADLMS subscore, and EWB subscore using SPSS Statistics (IBM Corporation, Armonk, NY). ANOVA was calculated for minutes used across all educational levels and across all vision statuses. Paired *t*-test and *P* value was calculated between initial and follow-up total, ADLMS, and EWB scores using SPSS Statistics.

## Results

### Patient Demographic Information

A total of 79 subscribers agreed to participate in the study and complete the initial IVI-VLV survey to assess vision-related quality of life. Ten participants were excluded due to having never received the Aira system or canceling before 3 months of follow-up. The final study group consisted of 69 participants (mean age, 52.1 years; range 20–82; 35 female and 34 male). Participants' highest educational levels were recorded (11% high school, 12% some college, 38% college, 28% masters or graduate degree, and 11% doctorates). The majority of participants had completed at least a college-level education. Participants' vision status (12% <20/200, 26% light perception only, and 62% total blindness), monthly subscription level (54% 100 minutes plan, 21% 200 minutes plan, 9% 400 minutes plan, and 16% unlimited minutes plan), and current use of assistive devices (white canes 61%, guide dogs 30%, or both 9%) were recorded. Mean time to follow-up was 108 ± 20 days. Mean total minutes used over the interval period was 334.1 ± 318.5 minutes. Services used include reading, navigation, home management, social interactions, shopping, instructions, and employment assistance.

### Improved Total IVI Scores, as well as Subset Scores Were Seen in Individual Using Aira

We asked whether the IVI-VLV scores using the Aira survey increased in patients between initial intake and 3 months afterwards. Paired *t*-test results for total, ADLMS, and EWB scores are shown in Table 1. Total score, as well as ADLMS and EWB subscores all were significantly improved from baseline (*P* < 0.0001).

**Table 1:**
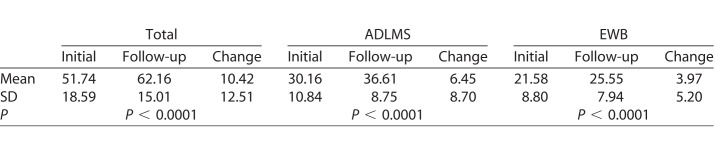
t-Test Between Initial and 3-Month Follow-up for Total, ADLMS, and EWB Scores

### No Correlation Existed Between Total Number of Minutes Used and IVI Score

We next asked whether the number of minutes used by individuals was correlated with their IVI score. Pearson correlation coefficients between minutes used during the 3-month interval and total, ADLMS, and EWB score changes were performed for this cohort (Table 2). There was no statistically significant correlation between minutes used and total score change (*P* = 0.098), and ADLMS (*P* = 0.055), and EWB (*P* = 0.051) subscores.

**Table 2:**
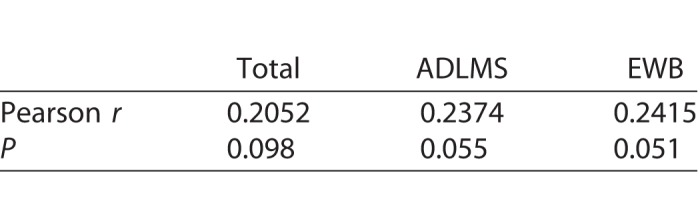
Pearson Correlation Between Total Minutes Used and Change in Total, ADLMS, and EWB Scores

### No Correlation Existed Between Total Number of Minutes Used and Education Level

Analysis of variance (ANOVA) was used to determine whether the number of minutes used by individuals was correlated with their educational levels. There was no difference in minutes used between all educational levels (*F* = 0.49028, *P* = 0.742826).

### No Correlation Existed Between Total Number of Minutes Used and Vision Status

ANOVA was performed to determine whether the number of minutes used by individuals was correlated with their vision status. There was no difference in minutes used between all vision levels (*F* = 1.75529, *P* = 0.182575).

### Average Net Cost Per Year is Lower Across All Subscription Levels Compared to Guide Dogs

We next asked whether the net cost per year was greater or less than that for guide dogs, given Aira's navigation ability. Over the 8-year assumed working lifespan of a guide dog, we calculated the 8-year projected cost for all subscription levels using previously studied cost-benefit values.^13^ Compared to the net cost per year of a guide dog ($2379), the price per year for unlimited ($1252), and 400 minutes (−$308), 200 minutes (−$1148), and 100 minutes (−$1628) plans was lower for Aira (Table 3). Based on subscription data, the average cost per point improvement in IVI-VLV score was $40.18 ± $203.40 over the 3 months.

**Table 3:**
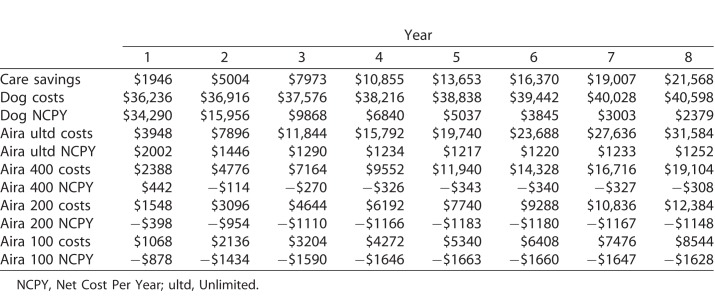
Net Cost per Year of Guide Dogs and Different Aira Subscription Levels Over 8 Years

## Discussion

This exploratory study assessed the quality of life of individuals living with SVI after using the Aira system. Among the 69 participants, the total, as well as ADLMS and EWB subscores exhibited significant improvement regardless of total minutes used. As a novel assistive technology for the SVI community, this suggests that the Aira system may improve quality of life for low vision patients and that Aira could serve as a useful instrument in the lives of those living with SVI.

Currently, assistive technologies designed for SVI individuals rely upon the remaining senses, or magnifying viable areas of the retina. For example, braille allows for tactile adaptation to reading, while audible traffic signals allows for auditory notification and direction. Despite this array of assistive technologies, many still are confined to narrow and specific applications. To expand beyond this, there has been increasing interest in co-opting existing mobile systems and networks including cell-phones. However, many of those experiments use an automated algorithmic system yielding only narrow intelligence with sparse nondynamic applications at this stage.^14^ These specific applications typically revolve around navigation and have, for example, used built-in acoustic or haptic feedback from vibratory motors of cell-phones^15^ and examined movement patterns to optimize waypoints in static environments, such as a house.^16^ Future applications have progressively continued to build around navigation, vertically stacking information related to that point of interest.^17^ Ramadhan^18^ described a similar wearable technology with GPS integration and ultrasonic sensors for feedback into three outputs of sound, vibration, and SMS output to monitoring individuals. Despite the similarity with Aira regarding feedback, the system Ramadhan describes only provides output to monitoring individuals after the adverse events occur during navigation, thus lacking the real-time assistive feedback provided in the Aira system. Beyond navigation, Neto et al.^19^ described a Kinect (Microsoft, Redmond, WA)–based face recognition system for visually impaired individuals with accuracy rates reaching 98.7% in optimal conditions. Despite the innovative use of existing technologies, this application remains confined to the single application of facial recognition.

Instead of relying on computed narrow artificial intelligence (AI) or unidirectional output to dictate assistance, Aira allows for overlaying of human–human intelligence, allowing for a cooperative approach towards issues commonly encountered by SVI individuals. This augmented reality approach allows for a broad spectrum of tasks to be accomplished from the classic navigation issue to complex musical lessons, which has resulted in a significantly increased quality of life.

With the emergence of many exciting technologies being applied as low vision assistive technology, the question remains whether these systems actually are useful to SVI patients. Thus, it is important to conduct clinical studies to rigorously assess whether these assistive technologies actually improve quality of life. Though patient-reported questionnaires have their limitations, they remain a useful metric to assess efficacy and use for these systems, especially as empiric metrics specific to vision may revolve around irrelevant measures in those with vision impairment, such as visual acuity improvement. Several biases may be present while undergoing self-report, including social desirability, recall bias, and selective recall.^20^ Recall bias is especially relevant to the IVI-VLV as it asks the participant to reflect on events of the past month. However, because on follow-up, the participant is again asked to use the most recent month as reference, this may serve as a temporal control to responses. Furthermore, concordance with recorded administrative empirical data is higher when recalling within the month compared to within the year.^21^ Patient-reported questionnaires are especially useful for responses that cannot be measured by objective data collection methods, thus self-report may serve as a suitable endpoint.^22^ The IVI-VLV, developed by an Australian project to assess the quality of life in those with severe vision loss, has been recommended for use by multiple trials including those studying the Argus II Epiretinal Prosthesis (Second Sight Medical Products, Inc., Sylmar, CA) and Retina Implant Alpha AMS (Retina Implant AG, Reutlingen, Germany).^12,23^

To our knowledge, this is one of very few studies to attempt to assess patient quality of life in low vision assistive technologies. In a clinical trial of the Argus II epiretinal prosthesis for patients with end-stage retinitis pigmentosa, the Vision and Quality of Life Index (VisQoL) was used to assess changes in quality of life after implantation of the Argus prosthesis.^12^ They demonstrated statistically significant improvement in three of the VisQoL subcategories after implantation. One difference between the VisQoL and the IVI-VLV survey is that the VisQoL was not validated in patients with profound vision loss and was not designed specifically for patients with SVI. Indeed, the IVI-VLV survey was not developed and available until after this trial began.

One interesting finding is that there was no correlation between the amount of minutes used and changes in score. Three reasons may explain the relationship, whether significant or nonsignificant, between number of minutes used and score improvements. First, the ADLMS (*P* = 0.055) and EWB (*P* = 0.051) subscores trended towards significance; thus, perhaps with more users, a significant correlation may have emerged. Second, this finding suggested that improvement in quality of life may have been due to psychologic effects of having a “safety blanket” in that they had access to the technology, rather than strictly from the use of the device. Despite many Cochrane reviews and studies analyzing the role of assistive technology in health outcomes, very few address this psychologic effect of the presence of the technology.^24–28^ In a Cochrane review of interactive health communication applications, the investigators suggest that significant efficacy of this technology is based on the cooperative combination of objective information with provided subjective social, behavior change, or decision support.^25^ Danilack et al.^29^ examined chronic obstructive pulmonary disease (COPD)–related reasons for patients not walking and showed that those who expressed more psychologic burden of their disease significantly had lower daily step counts. They concluded that reassurance may decrease the trepidation of those with COPD and enhance feelings of safety; thus, translating into functional outcomes.^29^ The same group studied 239 veterans with COPD who were randomized to receive a pedometer combined with an internet-mediated program or a pedometer as the control group. At 4-month follow-up, the intervention group had a significant increase in health-related quality of life. They concluded that psychologic factors, including fear and confidence, may be variables that are not addressed appropriately by nonresponsive technology, suggesting just the presence of the device does not confer equivalent benefits as with combining the device with active intervention.^30^ These reports provide valuable insight into the question of a placebo effect of the presence of Aira and instead demonstrate that the actual support the service provides may result in significant increases in quality of life. In a study by Papdopoulos et al.,^31^ depressive symptoms were correlated with less positive practical support in SVI individuals. Furthermore, visually impaired adults without any form of support displayed the highest levels of depressive symptoms, while those who received positive support displayed the lowest levels.^32^ In summary, it is reductive to assign the origin of improvement to only either objective support from the service or a subjective psychologic “security blanket” phenomenon. Thus, as a third explanation of the relationship between number of minutes used and score improvements, Aira and other similar assistive technologies provide a complex interaction of the two that combine to fully optimize the support given to the user, resulting in significantly increased quality of life measures. In our study, the lack of correlation between minutes used and score change may have not been due to a “security blanket” phenomenon, but from either the number of participants or the follow-up study period, though it is difficult to appropriately parse the true reason.

It is challenging to understand the interplay between providing enough support to meet those with SVI at their functional level and avoiding underestimation and overprotection.^32^ Though a mean of 92.8 minutes were used per month over the 3-month span, it will be interesting to analyze the trends of minutes used per month as users progress beyond 3 months either as they use more minutes as Aira enables them to explore more or as they use less minutes as Aira instills confidence to perform future tasks without assistance.

In addition to recognizing the benefits of the Aira system, it is important to place those benefits in the context of the actual costs to understand the net cost to the user and their environment. Using data from a cost-benefit study by Wirth & Rein in 2008 for guide dogs, a preliminary cost-benefit analysis was performed for Aira using figures of formal and informal care costs in the respective study that were extrapolated from the Medical Expenditure Panel Survey.^13^ Similar to our user population, the investigators analyzed the costs for those with visual acuity in the best-corrected eye of 20/200. Compared to the linear increases of total costs of Aira, guide dog costs followed a logarithmic function, though with a high initial cost of over $30,000 and a limited working lifespan. Thus, Aira not only offers a lower economic barrier to entry, but also when compared to the 8-year working lifespan of a guide dog, offers lower net costs every year regardless of subscription level. Compared to the host of functions Aira provides, guide dogs provide only a rudimentary form of navigation. With guide dogs, the user still must know turn-by-turn where they must go, and the guide dog enhances only the transit. With Aira, the user does not need to know the directions and instead can be assisted by an agent while retaining the transit enhancement. Beyond navigation, there is anecdotal evidence of guide dogs providing emotional benefits, though no formalized studies have addressed this impact to our knowledge.^33^ Similarly, in our study, we observed significant increases in EWB regardless of minutes used. As no studies have analyzed the quality of life improvements in assistive devices and technologies, this only provides a preliminary cost-benefit analysis without allowing for head-to-head comparisons.

At the time of the study, users were provided with first generation Aira smart glasses, which allowed for user interaction solely with the human agents through an AR overlay. With the release of the next generation Aira Horizon (Aira Tech Corporation) in early 2018, there is now a built-in AI platform called “Hey Chloe,” which provides voice-first technology, similar to interacting with Amazon Alexa (Amazon.com, Inc., Seattle, WA), and text recognition. Furthermore, as AI and use cases continue to expand, new functions currently are explored around a general suite of services, including Amazon Lex, Amazon Web Services (AWS) Lambda, Amazon Simple Storage Service (S3), Amazon Rekognition, AWS Internet of Things (IoT), and AWS Mobile Hub. Briefly described, Amazon Lex is a platform for voice or text conversational interface, AWS Lambda is an event-driven platform that executes functions in response to events, Amazon S3 is a cloud object storage platform, Amazon Rekognition is an image and video analysis service with integrated deep learning technology, AWS IoT is a bidirectional communications platform between AWS Cloud (Amazon.com, Inc.) and devices connected to the internet, and AWS Mobile Hub is central hub for AWS service configuration. The benefits of integrating this suite of tools as well as responsive AI into the future of assistive technology and specifically Aira include increased consistency and quality of AI-user interactions as well as the ability to scale, cost reductions, and diversion of dedicated human agents away from automated services to tailored interactions unavailable through AI. Future studies could investigate if this improved AI platform leads to increased improvement in quality of life in this SVI population compared to the first generation version.

Limitations of this study include the short follow-up time and the subjective basis of the patient questionnaires. In additional Aira explorers likely do not represent the demographics of all SVI individuals. These are individuals who have the financial resources to purchase a subscription to the service as well as the technical competency to use the device. Future studies would benefit from long-term follow-up to examine the presence and durability of benefits and potential disadvantages that arise from prolonged use of the Aira system.

In conclusion, this exploratory study shows that, regardless of minutes used, use of Aira significantly improves IVI-VLV total score, and ADLMS and EWB subscores for the severely visually impaired. This suggested that Aira may serve as a useful assistive technology to improve quality of life in SVI individuals, and further studies are required to assess the long-term benefits of this technology.
